# Exploring Definitions and Prevalence of Verbal Sexual Coercion and Its Relationship to Consent to Unwanted Sex: Implications for Affirmative Consent Standards on College Campuses

**DOI:** 10.3390/bs8080069

**Published:** 2018-08-02

**Authors:** Brandie Pugh, Patricia Becker

**Affiliations:** Sociology and Criminal Justice, University of Delaware, Newark, DE 19716, USA; beckerp@udel.edu

**Keywords:** campus sexual assault, verbal sexual coercion, freely given consent, perpetration tactics

## Abstract

Campus sexual assault is a pervasive issue impacting the well-being, quality of life, and education of all students. There have been many recent efforts to prevent and address campus sexual assault, most notably the adoption of affirmative consent standards. (1) Efforts to address sexual assault on college campuses through an affirmative consent standard could be undermined by traditional gender norms, sexual scripts, and the power dynamics inherent in heterosexual relations, which lead to situations in which many women provide consent to unwanted sex. (2) Studies indicate that college women are likely to experience verbal sexual coercion, yet research has failed to come to a consensus on how to define, operationalize, and study verbal sexual coercion. (3) Research on sexual consent is also lacking, in particular as it relates to consent to unwanted sex as a result of the presence of verbal sexual coercion. (4) This article discusses how multiple forms of unwanted sex can be conceptually examined. (5) Policy implications and areas for future research are discussed.

## 1. Introduction

Campus sexual assault (CSA) is a pervasive social problem widely known by college administrators, educators, law enforcement, government, and, most of all, students. In recent years, media attention has shined its spotlight on campus sexual assault. Headlines exposing the failure of college campuses to prevent and respond to campus sexual assault [1] have proliferated. Campus sexual assault has been labeled an “epidemic” [2], citing the high prevalence of sexual victimization found in studies. Perhaps due to this widespread attention, in recent years, there have been increased efforts to address campus sexual assault, including the previous White House Administration identifying it as a national priority [3,4,5,6,7,8,9]. Nonetheless, research suggests that a number of gaps in evidence-based programs and policies remain [6]. The emphasis on addressing campus sexual assault, the failure of universities to adequately combat and respond to sexual assault, and the focus on consent as central to address campus sexual assault [10], have led campus administrators to adopt affirmative consent policies.

The notion of affirmative consent has been around for some time now [11], but has really only gained momentum in recent years. Affirmative consent has been proposed as a way to remedy the issues with consent and to empower women and men in a dialogue of “communicative sexuality”, meaning that the sexual initiator (typically the man [12,13,14,15]) has to obtain verbal permission for each sex act from the person they are pursuing [16,17,18,19,20]. This effectively shifted sexual assault policy from a no-means-no standard to a yes-means-yes standard [20]. While this may seem like the most ideal response to CSA, an affirmative consent standard suffers from the same critique as other CSA policies that have not been carefully empirically or critically evaluated [6,21]. For instance, adopting an affirmative consent standard operates on least three major assumptions: (1) men, as the primary initiators of sex, will seek affirmative consent; (2) all women feel that they can freely say no to sex that is not wanted; and (3) initiators will take refusals seriously and respect them by ceasing all initiation of and requests for sexual activity. However, as will be argued throughout this paper, the research presented below primarily addresses the latter two assumptions and highlights that these assumptions do not align with a vast amount of empirical evidence. That is, in a substantial number of heterosexual encounters on college campuses, women’s choice to willingly engage in sex is thwarted many times because not all initiators take no for an answer. This begs the question as to whether an affirmative consent standard, which hinges entirely on providing women with that choice, would be effective in reducing, preventing, or responding to instances of sexual assault on college campuses [22,23].

### Research Gaps This Paper Seeks to Address

There are several gaps in the literature that this paper seeks to address, but first there are several things we would like to note. First, to cover a wide range of literature, we sacrificed separate in-depth analyses of sexual coercion and consent in order to focus more on breadth and tie these two concepts together conceptually. Due to the wide berth that this paper covers, we perceive this as a first step in a series of additional empirical, conceptual, and theoretical work. As such, there are several topics in this paper that we are addressing, if only to set the stage for future work. Second, in regards to sexual assault, men are primarily perpetrators and women are primarily victims [24,25,26,27], which is why we chose to focus specifically on sexually coercive tactics committed by men against women. However, we tried to avoid using the general term of “men” and “women” in describing incidences of sexual assault perpetration and victimization as not all men and all women are perpetrators and/or victims. Third, the majority of research has focused on heterosexual, white undergraduate women [5] and these are the current paper’s parameters for assessing CSA. Fourth, this paper included psychological, social, and legal scholarly work, however, in this paper there is an intentional focus on legal scholarship. This is due to the fact that legal notions of coercion, consent, and rape have influenced social and psychological work in these areas and also prevention policies. For instance, research on rape acknowledgment often describes that even though women’s experiences meet the legal definition of rape, they do not identify as rape victims [28,29,30,31]. Additionally, affirmative consent standards were initially outlined in legal work [10,32]. Last, it is not our intention to discuss the psychological consequences of different types of sexual victimization and to infer that certain types are more or less harmful. Psychological consequences to different forms of sexual assault (i.e., forcible rape, incapacitated rape, and verbal coercion) have been assessed elsewhere [33]. The main points of this paper are outlined below in Table 1. Table 1 shows the four gaps in the literature we have identified for this paper and how we plan to address these gaps.

Following the above sections, future research directions and concluding remarks will be discussed.

## 2. Sexual Coercion

Despite scholars moving in the direction of attempting to examine broader forms of sexual victimization and perpetration tactics, sexual coercion is understudied [56], producing definitions and operationalizations of sexual coercion that suffer from lack of consistency and theoretical conundrums [38,57,58,59]. These issues are partly due to the fact that different types of tactics used by perpetrators to obtain sex with unwilling partners have not been adequately defined or measured [59,60,61]. By using victim reports on unwanted sexual activity experienced by victims [34,38,46,56,62,63,64,65], the methods used by perpetrators to obtain sex from unwilling partners [37,45,59,66,67,68,69,70,71], or studies examining both victimization and perpetration behaviors [39,72,73], sexual coercion has been defined in several ways.

### 2.1. Sexual Coercion: Operationalizations, Definitions, and Conceptual Arguments 

Many scholars use the terms sexual coercion and sexual assault interchangeably or that sexual coercion encompasses all types of perpetration tactics that lead to sexual assault [59,74]. In these studies, sexual coercion is defined as a continuum of tactics to elicit sexual activity from unwilling partners ranging from non-forceful verbal tactics to physical force, with taking advantage of women due to voluntary or administered alcohol and/or drug intoxication somewhere in the middle [26,34,36,37,39,40,43,44,46,66,71,75,76,77,78,79,80]. Sexual coercion has also been specifically defined as tactics used following a partner’s refusal to sexual advances [36,46,48,70,81]. Others scholars treat sexual coercion as distinct from forcible rape, but not as a continuum [24,33,56,82] or as a construct entirely separate from sexual assault [38,83]. Sexual coercion has also been combined with physical force into one dichotomous variable [39,63,73,84,85]. In other cases, sexual coercion is equated to unwanted sexual intercourse, typically as the result of compliance or acquiescence [75,86] and sometimes is not defined as rape [26,61,83]. Similarly, one other study studied women’s consent to unwanted sex as women’s nonagentic sexual interactions (i.e., sex against their will) and stated that these situations were still technically consensual and were neither sexual assault nor indicative of healthy sexuality [50].

Most pertinent to the current article, many studies have begun to focus specifically on the idea that sexual coercion refers only to the use of verbally coercive tactics, rather than physical tactics [36,38,87]. Other scholars that endorse that sexual coercion is a continuum of tactics have begun to focus specifically on the tactic of verbal pressure [32]. This tactic is typically labeled as “verbal sexual coercion” (VSC) or “verbal coercion” (VC) and has been defined as the psychological pressure to engage in coerced sex [35,38,40,44,49,56,82,88] in the absence of physical force or explicit threat of force [89,90]. In the past, verbal coercion has been labeled “heterosexual coercion” [91], as well as “social coercion” and “interpersonal coercion” [44,92]. Additionally, researchers have stated that consensual sex that is unwanted or undesired as the result of compliance is separated from incidences of consensual sex that result from a partner’s coercive behavior [93]. In this way, they have separated coercive sex from unwanted or undesired sex [46,57,94,95]. Despite a relatively recent empirical focus specifically on VSC, not only are definitions, methodologies, and operationalizations of verbal sexual coercion inconsistent from study to study, but more nuanced studies examining the context, such as timing and tone of verbal coercion, have been lacking [32,56]. 

Furthermore, much of the research on VSC has only examined a few factors that constitute VSC, such as continual pressure, showing displeasure, or using authority to obtain sex from an unwilling partner, particularly those studies that have utilized the Sexual Experiences Survey, either the original, revised, or a modified version [35,36,37,43,49,62,71,82,83,94,96]. More nuanced studies have been conducted to examine a wider range of verbal tactics to obtain sex from an unwilling partner [34,38,67]. Studies on verbal sexual coercion typically examine tactics such as nagging or trying to convince, ignoring, threatening to break up, and to more forceful forms of verbal coercion such as displaying anger or yelling [34,38,46,49,56,75,88]. In addition, one group of researchers found a difference between a partner’s use of benign, seduction tactics (i.e., sexual coaxing), and VSC (e.g., verbal pressure) [67]. Similarly, other studies have found that trying to arouse a partner with sexual touching or compliments is a common way to obtain sex from an unwilling partner, but these behaviors are decidedly different from verbally coercive tactics [46,48,67,75].

In this paper, we are primarily utilizing the term verbal sexual coercion and defining it as a tactic utilized by a perpetrator in a sexual encounter to persuade or coerce the other person to agree or give in to sexual activity “against freely given consent” [84] (p. 99) and is distinct from situations in which women consent to unwanted sex without the presence of VSC [46,57,95]. While the involvement of alcohol and/or drugs, verbal threat of force, or physical force could be present in verbally coercive situations [34,50,56], we are primarily interested in discussing verbal sexual coercion in the absence of immediate victim incapacitation and/or a perpetrator’s use of physical force [89,90]. Recent research has also shown that variables representing manipulation tactics and substance use or force load onto separate factors, indicating that it may be useful to separate VSC from incapacitation and force as tactics to obtain sex from unwilling partners [38]. We attest, as with many other scholars, that verbal sexual coercion is just one tactic of sexual assault on a continuum ranging from non-forceful verbal tactics to incapacitation to physical force [26,34,36,37,39,40,43,44,46,66,71,75,76,77,78,79,80]. There are many reasons articulated throughout this paper as to why we believe that this conceptualization is necessary, but it is also worth noting that research has suggested that women experience similar levels of distress despite the type of perpetration tactic [97], suggesting the utility of including tactics of verbal sexual coercion on a continuum with other perpetrator tactics. The following section outlines the prevalence of unwanted sex as a result of VSC coercion on college campuses.

### 2.2. Prevalence of VSC on College Campuses

Feminist rhetoric and empirical evidence suggest that some campuses are rape-prone [98], where sexual assault is the norm rather than the exception. Research specifically focused on sexual activity resulting from VSC highlights the high prevalence of this form of sexual victimization on college campuses. Perhaps because of its high prevalence, college students have begun to recognize a wider range of sexual assault incidences that do not conform to the “real rape” stereotype [47]. For instance, while college students readily identify physically forced sex as rape and as unacceptable [86], recent research at one university also shows that they identify rape as something that can occur as a result of verbal pressure or coercion, or when one takes advantage of a person who is under the influence of drugs or alcohol [99]. This suggests that these college students’ perceptions about what constitutes sexual assault are in line with the reality of sexual assault on college campuses.

The majority of sexual assault on college campuses, as well as off campus, rarely involves physical force or additional physical injury [26,48,62,82], and instead is more likely to involve drugs and/or alcohol [51,52], and/or verbal sexual coercion with a substantial number of studies implicating verbal sexual coercion as the most common sexual assault tactic [34,35,36,37,38,39,40,41,42,43,44,45,46,47,48,49,50]. The use of alcohol in campus sexual assault has been extensively researched elsewhere [51,52] and is outside the purview of the current article. The prevalence of unwanted sex resulting from VSC varies as a result of numerous definitions of what constitutes coercion, the timeframe measured, and the type of sexual victimization assessed (e.g., completed sexual intercourse or a broader range of unwanted sexual contact). Additionally, some studies have used a limited number of items to capture sexual coercion while others have used substantially more [94]. Nonetheless, there is existing research that sheds light on the high prevalence of unwanted sex, particularly as a result of the use of VSC. 

A recent systematic overview on the prevalence of unwanted sexual contact ranging from any type of unwanted sexual touching to attempted or completed rape examined the prevalence of unwanted sexual contact resulting from verbal coercion (e.g., intimidation, pressure, lies, threats, or continual arguments). The authors reported that among college women, the prevalence of unwanted sexual contact as a result of verbal coercion ranged from 1.7% to 32% [5]. Although not restricted just to women in college, a recent study explored different ways of scoring the Sexual Experiences Survey (SES), which is one of the most common measures of sexual assault events [62,90]. In this survey women are asked if they experienced sexual contact, attempted or completed rape, without their consent as a result of a range of perpetrator tactics (i.e., verbal coercion, threat of force, physical force, and incapacitation) since the age of 14. This study combined two measures of verbal coercion: (1) telling lies, verbal threats, making promises known to be untrue, or using verbal pressure and (2) showing displeasure, criticizing, or getting angry. Fifty-eight percent reported unwanted sexual contact by coercion an average of 2.3 times, 55 percent reported attempted rape by coercion, and 46 percent reported completed rape by coercion [62].

One small-scale study that examined CSA through “social coercion”, which was similar to our definition of VSC, found that during college, 20% of women reported unwanted sexual intercourse through social coercion, compared to 7% as the result of forcible coercion [100]. Furthermore, another study found that 9.5% of women, while in college, experienced forcible rape, while 11.7% of women reported oral, anal, or vaginal penetration because of VSC, defined as continual arguments, pressure, or misuse of authority [82]. Another study on college students and high school students, both male and female, found that 30.5% reported that they experienced manipulation tactics as a way to get them to engage in sex that they did not want [38]. Furthermore, one study examining college women’s nonagentic consent to unwanted sex found that 32.9% of respondents reported one or more experiences of nonagentic sexual interactions and more than half (60%) of them reported two or more incidents. Of these incidents, women reporting that these experiences occurred while on dates, 22.2% reported that their date used threats, force, or manipulation and 18.3% reported that their partner ignored their protests [50]. Another tactic where a partner moved forward with sex before giving the woman a chance to protest was listed as a reason why women reported experiencing nonagentic sexual interactions [50].

Another study, defining sexually coercive behavior as the act of using pressure (e.g., trying to sexually arouse the other person, emotional manipulation, or lies), alcohol or drugs, or force to obtain sex with a person after they already refused to engage in sex, found that 78 percent of college women reported experiencing at least one tactic [48]. The most common tactics following sexual refusal were: trying to induce sexual arousal (73%), repeated requests (66%), threat of harm (66%), voluntary intoxication (42%), or being told lies (42%). A similar breakdown of prevalence by tactic was found by Katz and Tirone [46] in their study of 76 college women that were sexually active in relationships. A little over half (52.6%) of their sample reported one or more instances of their partner behaving sexually coercive by attempting to arouse (44.7%), emotional manipulating and deceiving (28.9%), intoxication (7.9%), and force (1.3%). Interestingly, 40.8% of the women in this study reported giving in and having sex even when they did not want to and for the vast majority of respondents, giving in was directly related to a partners’ previous use of sexually coercive tactics ranging from verbal coercion to physical force. Yet another study that directly captured whether or not college women consented to sex following VSC found that 60% of women gave in to their partners’ VSC, even after demonstrating non-consent through physical or verbal assertive strategies. Since these women voiced non-consent, they reported feeling powerless to stop their partner, which is why they decided to give in [34].

Studies on perpetration have found a range of prevalence of verbally coerced sexual intercourse. One study found that 17.2% of a community sample of young men reported verbally coercive behavior to obtain sexual intercourse [43]. Similarly, another study examining three items of verbal coercion (i.e., being unable to stop oneself, threatening to end the relationship, continually arguing, or saying things “you didn’t really mean” to obtain intercourse) found that 20% of college men endorsed at least one of these behaviors [35]. A smaller percentage of college men in a longitudinal study reported utilizing verbally coercive tactics, 3.3% within an 8-month period, and 4.9% at the 1 year follow up [71].

Overall, there is a wide range of prevalence rates based on the sample (e.g., college students overall vs. college students in relationships), questions to ascertain VSC, timeframe, and whether or not questions were asked of perpetrators or victims. Nonetheless, it appears that between 3–20% of college men report using VSC to obtain sex from unwilling partners. Studies assessing victimization that have examined fewer items of VSC, have reported that 1.7 to 3.2% of women have experienced VSC. Studies with more indicators for VSC have found that between 40 and 60% of women answered that they have at some point compiled to unwanted sex as a result of past or current VSC.

## 3. Consent

Prior to illuminating definitional issues surrounding consent, it is important to first outline how we are conceptually framing consent as it pertains to VSC and societal pressure [101]. We contend that consent resulting from VSC is separate from consent from societal pressure or expectations [102,103]. Societal pressure or expectations refers to broader social norms that teach women to have sex out of duty or to be available for men’s pleasure, among others [101,103,104,105]. For instance, women that consented to unwanted sex were more likely to endorse beliefs in the traditional gender roles, which was related to finding reasons to consent to sex [105]. Although societal pressure is not divorced from VSC, research has articulated differences between consent arising from VSC and consent resulting from societal pressure [21,75,77,91,106]. In addition, we have done our best to explain consent and VSC separately, but consent is also closely tied to the incidence of VSC on its own and also because both the occurrence of VSC and negotiations of (non-)consent are heavily influenced by traditional gender norms, sexual scripts, and the campus hook-up culture [13,14,105,107]. In the following paragraphs, we will discuss various definitions of consent and the negotiation of (non-)consent in heterosexual encounters.

### 3.1. Definitions of Consent

Consent appears to be an elusive, under-researched topic [19,53] and consent is particularly understudied within the college context [32]. As Jozkowski [20] pointed out, it is concerning that there is limited research on consent [53] given that consent is typically the line between sex and rape and the majority of prevention programs focus on consent. Research on consent is also supremely important while campus administrators and lawmakers explore the potential effectiveness of affirmative consent policies. It has been argued that society has a male understanding of sexual consent, with women’s sexuality often being defined for them in the legal realm [47,108,109]. Perhaps because of this, legally, the term consent is often obscured in definitions of force and/or is ambiguous and unclear [17,110,111]. As a sociological or psychological concept, scholars demarcate between non-consent and consent. Non-consent ranges from the victim’s state of mind—“against her will” [112,113,114] to behavioral—saying no, fighting, crying, pleading for him to stop, or as silence [113,114]. On the other hand, consent is often thought to refer to shared and freely given agreement of entering into sexual activity [84,115,116,117]. This is also endorsed by the Centers for Disease Control and Prevention (CDC) as they define sexual violence as something that occurs without freely given consent and includes incidences of intimidation or pressure that resulted in an inability for the victim to refuse [118]. In sum, while we acknowledge that both non-consent and consent can be communicated verbally and nonverbally, we argue that consent should be freely given, free of VSC and force.

### 3.2. Negotiations of (Non-)Consent in Heterosexual Encounters

Two of the first studies on sexual consent in college were conducted by Hall [119] and Hickman and Muehlenhard [115], and both found that consent was communicated more frequently with nonverbal cues instead of verbal cues. Hall [119] found one difference in how consent was negotiated based on the sexual activity; verbal cues were used more often for intercourse and nonverbal cues for sexual touching. In a more recent study, Jozkowski and her colleagues [120] found that while men reported using verbal cues to communicate their sexual consent, they used nonverbal cues to interpret women’s consent. On the other hand, women reported that they use verbal cues to communicate consent, and used a combination of verbal and nonverbal cues to interpret their partner’s consent. In the following two subsections, we outline women’s (coerced) consent to unwanted sex as a result of VSC and some men’s responses to non-consent.

#### 3.2.1. Women’s (Coerced) Consent to Unwanted Sex as a Result of VSC

Traditional gender roles and sexual scripts ascribe that women are responsible for asserting their (lack of) desire in sexual situations and play the role as sexual gatekeeper [14,32,109,120,121,122,123,124]. Yet, the existence of the social script that women should avoid saying no, means that clear refusal in sexual encounters appears harder for women, especially those who more heavily adhere to gender- and sex-role stereotypes [105,122,125,126]. Gender roles encouraging women to defer to men may influence women to be more concerned about their male partner in these interactions than they are with their own autonomy [75].

Similarly, popular opinion is that women’s resistance to sex is ‘token’ [112], and that women want sex, but may initially refuse in order to save face and not be labeled ‘easy’ [127,128,129]. However, Shotland and Hunter [130] in a close analysis of token resistance suggested that women retrospectively may believe that their resistance was insincere, yet their lack of desire was sincere at the time and what happened was a change in intention. This change in intention could represent situations in which the woman realized that their partner was not going to stop the sexual activity and therefore, coupled with feelings of powerlessness and the desire to avoid the self-stigma and trauma of being raped or additional harm, she may “give in” [34,46,48,50,56,91,102,104,131,132].

This idea that some men do not take no for an answer is supported by research that has found that many women do indeed voice or physically indicate non-consent and some men use VSC or other tactics to obtain consent from her despite the sexual refusal. In fact, one study found that 81% of women who experienced rape through incapacitation, threats of force, or force, voiced non-consent before or during the rape [133]. Similarly, other studies found that women that complied with unwanted sex only did so after voicing non-consent or demonstrating physical resistance (which is a less common resistance strategy) [34,50,56,133]. These studies show that these men were aware of their partner refusing sex and not providing consent, but ignored the lack of consent and proceeded with utilizing coercive tactics to obtain sex [50,134].

Situationally, when unwanted but consensual sex is the result of VSC, this renders any additional tactics of coercion or force unnecessary [57,122,135,136]; if initial tactics were unsuccessful, other tactics may be used and some men will escalate to using physical force [40,56,67,70,75,76,137,138]. Some women report fearing the escalation of violence as one study found that some women reported consenting to sex out of fear of further physical aggression or fear of being raped [56]. This study also found that some men progressed to more severe tactics to obtain sex when their initial tactics were unsuccessful [56]. This fear response could also have been due to the fact that in some cases verbal tactics occurred in tandem with physical aggression [34,56].

This could reflect why there is a higher percentage of women that report consenting to sex that they do not want than the percentage of those that report physical force and why there is a higher percentage of men that self-report the use of coercive tactics rather than physical force [67,81,84]. This suggests that physical force is unnecessary since many times women comply with unwanted sex as a result of VSC. If a man is clearly disregarding the woman’s lack of consent and continuing to pressure or coerce her into engaging in sex, she may believe that there is nothing she can do to stop the behavior. Therefore, women may rightfully experience both powerlessness and fear more violence and therefore “submit to survive” [139] (p. 177). While these are situational responses, consent in these situations could also be related to historical experiences with that partner or previous partners.

Katz and Tirone [46] found that a large majority of undergraduate women in their sample reported consenting to a partner’s request for sex based on previous patterns of coercion to obtain consent. Another study found that 20% of women in their sample said that they gave in to sex because they feared the reaction of their partner [94]. Similarly, one study found that past negative sexual encounters were related to sexual compliance, suggesting that this could be because they learned that not submitting resulted in greater negative consequences. This study found that 81 percent of women reported multiple sexual victimization experiences [62]. Another study found that women that were more likely to comply with unwanted sexual advances were also more likely to report emotional discomfort during their first sexual encounter, suggesting that historical experiences influence their compliance to future unwanted sexual advances [123]. Yet another study found that women directly attributed their consenting to unwanted sex due to previous sexual trauma they experienced, despite this question not being part of the research design [140]. It is possible that women who have experienced previous sexual victimization are more likely to believe male aggression is normal and therefore consent to unwanted sex [131].

As early as the 1970s and 80s, scholars suggested that women may consent to unwanted sex so they will not be raped [77,106,112]. Research from contemporary scholars appears that preventing further harm or sexual assault is indeed a major reason why women consent to sex that they do not want [34,125]. In this way, power dynamics at play in heterosexual relations where some men do not take no for an answer and where women are taught to defer to men from the outset, create situations in which women do not feel that they can say no to unwanted sex [104,132]. Women tend to feel the ramifications of this, as research has shown that many female victims blamed themselves for not saying no, for not being more adamant in their sexual refusals, or for complying [31,34,49,75,141].

While unlikely to identify coercive experiences as rape [30], many women report that these experiences still cause harm [34,48,50,56,140]. In one study, women who reported engaging in unwanted, but consensual sex, often identified their experience as ‘problem’ sex and implicated sexual coercion [142]. In situations where women consent to unwanted sex, women may engage in self-blame, or minimize, justify, or normalize the VSC they experienced [49,75,141]. Another study found that women that reported consenting to sex as a result of coercion experienced similar PTSD symptoms as the women who reported rape than the women who were not victims [49]. Similarly, women that reported experiencing or fearing physical aggression, which was also related to consenting to unwanted sex, experienced long-term psychological distress [56]. Overall, many women that consented to unwanted sex reported feeling guilt, anger, anxiety, and depression [140], as well as experiencing self-blame [50,75], regardless if they labeled their experiences as sexual assault and regardless of perpetrator tactic [97].

#### 3.2.2. Men’s Response to Non-Consent

Adams-Curtis and Forbes [84] discussed that one consistent finding in the literature on sexual assault is that self-reported perpetration prevalence is lower than self-reported victimization [32,143]. Despite this discrepancy, research on sexual assault perpetration, either directly from studies examining perpetrator behavior or from reports by those victimized, indicate that women’s interpretations of heterosexual situations, when freely given consent is not present, appear to be fairly accurate. Recall that we are not focused on alcohol-related sexual assault here, which tends to have its own protective and risk factors for perpetration [136], although some research has shown cases where there is a presence of both verbal sexual coercion and alcohol use [34,50,56].

Most of the time men stop once they have received a sexual refusal [89], however some men continue even after hearing sexual refusal [48]. These men may believe that “working a yes out” following sexual refusal is a perfectly acceptable way to obtain sex from unwilling partners [144,145,146]. It could be that men believe that women’s resistance was ‘token’. Token resistance beliefs were found to have a significant negative association with interpreting a sexual assault in consent scenarios (e.g., one partner pressured the other into having sex when he or she did not want to) [147]. In this study, both token resistance and rape myth acceptance had significant negative associations with interpretation of sexual consent in complex scenarios [147]. Interestingly, a protective factor to this was sexual communication assertiveness as this was positively associated with correctly perceiving sexual assault and sexual consent scenarios. That is, in cases where men reported that they are willing and able to engage in clear communication to obtain sexual consent, this was positively associated with interpreting sexual assault and sexual consent situations appropriately [147]. 

In this way, it could be argued that what happens following a sexual refusal is merely a miscommunication. However, instances in which men hear a sexual refusal, but do not take it seriously or ignore it, are not a matter of miscommunication as to the seriousness of sexual refusals [148]. For example, O’Byrne, Rapley, and Hansen [127] reported that men do understand when women are refusing sex, even in cases in which women refuse sex in subtle ways (as described in a previous paper [149]). Echoing this, Beres [146] found that men and women both are able to ascertain their partner’s consent based on nonverbal cues and context. In fact, researchers have also found that men are able to evaluate refusal regardless of whether or not it was provided verbally or nonverbally [150]. In this way, it is not that these men do not know what is happening, but they are deliberate in their actions. As an example, one study found that men who reported committing sexual violence in the past were more likely to say that they did not expect to respect and adhere to their partner’s decision to engage in sex or not [151]. In addition, reporting an interest in the use of VSC to obtain sex was correlated with reports of past sexual aggression [67].

In situations where some consensual activity has occurred, men may also believe that women are willingly consenting to all sexual acts, despite indicating non-consent or a lack of willing consent [148,152,153]. Some research has shown that sexual precedence (e.g., having previous sexual access to a partner) was related to the use of perpetration tactics to receive compliance [56]. Use of tactics in an intimate relationship may be related to overall pattern of coercion [46], having access to these women to use coercive tactics [56], and/or believing that they were entitled to have sex since they had sex with them before thereby justifying their use of coercion [66,154]. While it could also be argued that some men move forward with sex because they genuinely believe that the woman consents and desires sex, some scholars argue that this is due to a self-serving bias. For instance, men who report misperceiving women as wanting to engage in sexual intercourse were also likely to report using sexually aggressive tactics to obtain sex [37,155]. Similarly, men that were more likely to endorse rape myths reported greater perceptions of consent and desire [150]. Closely related to this are feelings of entitlement [66]. For instance, general and sexual entitlement influences rape-supportive attitudes, which in turn predicts sexual aggression [154].

This research indicates that at least most men are well aware when their partner is not consenting, yet some will persist with sexual activity because they do not care about the victim’s feelings [115] and/or because they believe that the more they persist, the more likely she will eventually give in [156]. This is without considering or caring that women give in precisely because men are persistent and are not taking no for an answer [48,56]. In a study on negotiations of sexual consent, Jozkowski and Peterson [120] discovered a small percentage of men who behaved intentionally deceptive in obtaining sex by stating that sometimes when initiating sex to a partner who objects, they ignore their objections and proceed with intercourse, and often justify their actions by claiming the physical intercourse happened “by mistake”. Some men also may completely forgo asking for consent if they know that the woman will say no [120]. In a study on women’s nonagentic sexual experiences, women cited that some men engaged in sex without giving them time to protest, proceeded with sex despite their protests, or used other manipulation, threat of force, or physical force tactics to obtain sex, and the first two tactics were more common than the last one [50]. Similarly, a content analysis of men’s online justifications for rape revealed some instances where men reported ignoring women’s protests and proceeding with sex [148].

Other men may escalate their level of VSC or resort to physical force if their initial tactics did not result in sexual compliance. Some men may ignore sexual refusals outright and proceed with forms of coercion or resort to force to obtain sex [66,75,76,134]. One study that separated types of sexual aggression (i.e., forced sexual contact, verbally coerced sexual intercourse, and attempted or completed rape), found that 48 percent of heterosexually active men reported only one type, 22 percent reported two types, and 30 percent reported three or more types of sexual aggression [43]. This indicates that in individuals with the propensity for sexual aggression, verbal sexual coercion and other forms of sexual assault perpetration could co-occur or one tactic could progress to another. In fact, a few studies have shown that men that verbally coerce their girlfriends into sex also may be more likely to have been physically violent in the past or are more likely to become physically violent in the future [67,137,157,158]. Other research in the domestic violence literature has found a correlation between psychological aggression and physical aggression over time [159,160,161]. Similarly, men who are in line with gender norms may associate sex with dominance, which could culminate into men acting in a coercive manner to obtain sex [162]. For example, men who use sexual coercion may be using these tactics as a way to establish and/or maintain a sense of power and control over the other person [81,148].

In conclusion, there is a substantial heterogeneity in sexual assault perpetration and many men do not sexually assault women [37,67,163]. However, some men do sexually assault women and such behavior is influenced by the heterosexual script that contends men are the ones to seek sex and women are the gatekeepers; this burdens women and simultaneously influences beliefs that these coercive tactics are legitimate [21,75,120,148]. In this way, negotiations of consent are interlaced with gendered norms and sexual scripts. The current legal (and arguably, social) definition of (non-)consent does a disservice to women because it fails to acknowledge the gendered power dynamics at play [53]. It is crucial that the legal realm and scholars examine how gender differences influence negotiations of consent through the role of power embedded in masculine and feminine social norms [164]. Furthermore, the difference in signals used by men and women to communicate consent and how these signals are influenced by sexual scripts and power dynamics [121,122] need take the utmost precedence. Gender roles, sexual scripts, and power dynamics all influence the action of and response to VSC, as well as the perceptions, interpretations, and the actual act of providing of consent, culminating in many cases of unwanted, but (perceived as) consensual sex. In the section below, we discuss the relationship between VSC and consent and we offer a conceptual paradigm to examine sexual compliance and coerced consent.

## 4. The Relationship between VSC and Consent

As stated earlier, the research on sexual victimization lacks a much-needed consensus on how VSC and its related outcomes should be categorized or studied. With all the various ways the tactics and outcomes of VSC are defined, measured, and studied, the primary concern of this paper relates to research that has argued that consent resulting from VSC is compliance or acquiescence and may be harmful consensual sex, but consensual sex nonetheless. It is not our intention to indicate which types of sexual assault perpetration tactics result in more or less psychological distress, but rather to articulate that how we conceptualize and study rape, and how we interpret consent resulting from VSC to have direct implications for policy, law, and how women and men understand these types of incidents [165]. For instance, coming to a consensus on how to interpret consent arising from VSC directly impacts future cases of sexual assault under an affirmative consent standard.

It is possible that one reason these issues exist is because the research has failed to adequately study and consistently define both sexual coercion and consent, and how these two are interconnected. In the following paragraphs, we will first outline the ways in which consensual but unwanted sex has been conceived as harmful consensual sex or as a harmless compromise in the context of relationships. Then, utilizing much of the evidence presented earlier in this paper, we summarize the argument that non-consent and a lack of freely given consent is present in cases of VSC, and that this should be identified as sexual assault. Finally, we tie these arguments together and provide a chart to illustrate our position on how these situations could be conceptualized.

### 4.1. Sexual Compliance

Neoliberal politics state that women are free to engage in the hook-up culture or exert their sexual power and desires [22,23,122,132]. This view sees women as sexual agents that may consent to desired sex as well as sex that is not desired for any number of reasons, suggesting that consent is freely given. In this way, sexual compliance is parallel to non-coercive sexual activity and is consensual sex which is distinct from rape [105,114]. West [103,166] described sex resulting from sexual coercion as harmful, but consensual sex, and should not be seen as criminal [167]. The idea is that consensual sex does not involve the same injury as nonconsensual sex (i.e., forced sex) [166], and rather than defining it as rape, scholars should recognize and examine the harms of consensual sex [104]. West [103], a well-known cultural feminist argued that consent to unwanted sex is a byproduct of societal expectations, as well as the erotic desire of women to experience submission to a domineering man [166]. Overall, this position contends that when women provide consent to unwanted sex, this may be harmful, but not necessarily equate to sexual assault [65,110]. After all, scholars do suggest a difference between “sexual wantedness” (i.e., desire for sex) and consent (i.e., giving permission to have sex) ([150,168], p. 73). For example, one may desire sex but not consent to it, while others may consent to sex but not desire it [114].

Along similar lines, other researchers have found that consent to unwanted sex can be a relatively harmless experience and occurs because of the desire to maintain and preserve relationships [101]. With this in mind, sexual compliance could simply be a compromise or sacrifice in relationships akin to consenting to other activities in a relationship that one does not want to do (e.g., going to a movie or a dinner place that is not desired) [95,169]. One study found that women who engaged in consensual but unwanted sex in the context of relationships, found that these situations were absent direct tactics by the perpetrator to obtain sex and were not associated with any negative consequences, despite indicating that their partner was aware of their lack of desire. These participants instead reported positive consequences, such as greater partner satisfaction and relationship intimacy [95]. Similarly, a longitudinal study on sexual compliance found that some women complied with sex later in the relationship without reporting partner use of VSC, indicating that some women would consent to unwanted sex in the absence of VSC [46], perhaps in order to preserve the relationship [101]. Even the presence of coercion does not necessitate a negative response as a small number of women in Jeffrey and Barata’s [75] sample cited some positive aspects to their experience of coerced sex. These women reported that they felt closer to their partner after the incident either by learning more about what he wanted sexually or by the conversation that took place as a result of the incident.

It is not a radical idea to assume that having sex in the context of a relationship is something desired by many people who decide to enter into intimate relationships and therefore could be requisite for maintaining a relationship [114]. Some people may desire emotional intimacy over physical intimacy or have a lower sex drive than their partner, but could agree to have sex in the absence of desire to please their partner [95,150,168,169]. As long as this happens without VSC or other perpetration tactics, then it is conceivable that this is freely given consent and simply a compromise to maintain the relationship [101,170].

While it is clear that women (and men) may consent to sex that is unwanted and without VSC, as a normal part of intimate relations either independently or because they adhere to societal expectations (e.g., it is one’s duty to have sex with your partner), these situations are distinct from consent that arises from the situational and/or historical use of VSC [46,57]. Focusing on consent as the demarcation of rape and consensual sex while ignoring the situations of how consent came about is problematic [53]. This not only reinforces the notion that women can accept or deny sexual relations without repercussion, but it also ignores the power dynamics at play in heterosexual encounters and the fact that women do not feel that they can say no to unwanted sex [22,34,53,56,57,104,132]. It should be noted that in these situations, women feel like they cannot say no to sex precisely because they already did say no, but to no avail [34,56,82]. It is precisely this argument that supports the idea that consensual sex resulting from VSC is not freely given consent and this is important given that an affirmative consent standard rests on this assumption.

### 4.2. Consent Resulting from VSC Is Not Freely Given and Therefore Akin to Sexual Assault

It is conceivable that the focus on consent as the demarcation between rape and sex [105] has rendered consensual sex resulting from VSC a gray area, while force and resistance (and therefore unambiguous non-consent) is black and white. Verbal pressure by men has historically been interpreted as legal or extralegal seduction, leading to reluctant consent, but consent nonetheless [110]. For instance, some scholars have decidedly separated sexual coercion from rape, based on the presence of force (or alcohol use) and elements of consent [26,82,83,105,166]. What is more, the historical focus on rape as nonconsensual sex that involves force and resistance coupled with more recent rhetoric of sexual empowerment and sex positivism has muddied the waters on the relationship between sexual coercion, consent, and rape. This is because neoliberal politics stating that women are free to engage in or decline sex emphasize the consensual aspect of consensual but unwanted sex. At the same time, the majority of studies on consensual, unwanted sex have rarely discussed both the idea of sexual compliance and consent not freely given as a result of VSC, nor have they critically examined the relationship between VSC and consent.

While scholars in the literature on interpersonal violence have been expanding definitions of interpersonal violence to include psychological tactics [171], some research has decided either not to consider sex resulting from VSC as sexual assault or as non-criminal sexual victimization [26,61,83,113]. Yet, it can be argued that VSC to obtain sex from an unwilling partner is akin to psychological warfare that is termed abuse in the domestic violence literature. It is our opinion that, based on the information presented thus far, that there is a disconnect between the failure to acknowledge sex resulting from coercion as rape and feminists’ emphasis on the notion that rape is something that can occur without force and between people that know each other [172]. On the one hand, scholars identify that rape does not typically involve force and the presence of force or threats of force are not necessary for an incident to be considered rape. On the other hand, some scholars argue that sex that involves coercion is not rape, especially if the woman consented. The former focuses on the absence of consent as the primary defining characteristic of rape. The latter also focused on this, but instead contends that since consent is present, it is not rape. Some scholars argue that even though this type of unwanted sex is consensual, it is not without harm [103,104]. While this may be the best way to conceive of this type of unwanted sex, it fails to acknowledge many of the findings presented above. In the paragraphs below, we argue that the position that sex resulting from VSC, especially when consent is present should be considered distinct from rape, fails to take into account at least two factors: (1) coercion would not need to be present if consent was given initially and (2) consent as a result of VSC is not freely given.

#### 4.2.1. VSC (and Force) Would Not Be Present If Consent Was Given Initially

MacKinnon argued that force is present in sexual assault scenarios precisely because there was a lack of consent [106]. It is in our purview that VSC tactics are also present because of initial sexual refusal [48,57,122,135,136]. Feminist scholars argue that verbal sexual coercion is fueled by invisible power dynamics that justify men’s use of coercive tactics, prohibit women from making free and autonomous decisions on when, how, and where to engage in sexual activity, or utterly obscure the process by considering it to be a normal part of heterosexual relations [66,75,84,104,154]. Recall that Cook and Messman-Moore [133] found that a large percentage of forcible or alcohol-related rape survivors reported that they voiced non-consent either before or during the sexual assault, findings echoed by other reports of unwanted sex as a result of VSC [34,56]. Along these same lines, Struckman-Johnson and colleagues [48] found that a large percentage of women were subjected to at least one form of sexual coercion following sexual refusal. A third of men reported using more than three tactics of sexual aggression, suggesting that they either used different tactics on different people or the same person, or progressed to other tactics over time or within the same encounter [43]. Other times, men may simply engage in sex without obtaining consent, while others ignore protests, or resort to additional tactics, which includes physical force, and continue with sexual activity [34,40,50,56,67,70,75,76,134,137,138,144,145,146,148,157,158]. Women may also comply to unwanted sex based on historical patterns of coercion even if at the time they complied no perpetration tactics were used [46].

This suggests that a substantial number of women give in to sex when men are already not taking no for an answer. Women may rightfully fear experiencing further violence or sexual assault and therefore give in so that they can avoid additional harm and/or perceived negative personal consequences of sexual assault [34,46,48,50,56,91,102,104,131,132]. In these situations, these men may wrongly assume that they received consent, when what happened instead was that their partner felt they did not have a choice to decline sex [56] precisely because their initial sexual refusal was not respected [36,46,48,70,81]. When it is unlikely that resistance would or did result in stopping sexual activity, the responsibility for unwanted sex should be placed on the perpetrator and not the victim, especially because the victim did not provide freely given consent [88]. Recall that the CDC defines sexual violence as a sexual act without freely given consent against someone that includes instances where the victim was unable to refuse because of the presence of intimidation or pressure [118]. 

#### 4.2.2. Consent as a Result of VSC Is Not Freely Given

We argue that the literature in the sections regarding women’s consent to unwanted sex as a result of VSC and studies on men’s response to non-consent, and the fact that VSC would not be present if consent was given initially, should be enough to conclude that consent resulting from VSC is not freely given. Bay-Cheng and Bruns [140] in their study of unwanted, but consensual sex, questioned whether sexual compliance should be considered freely given consent. With the backdrop of the broader culture, both in society and on campus, situations where consent results from VSC have to take into account the perpetrator’s actions as well as the power dynamics inherent in heterosexual relations and the society that only condemns, even if rarely prosecutes or convicts, perpetrators in cases of unambiguous sexual assault.

Moving toward this paradigm of how to view instances of consent resulting from VSC is similar to how scholars and the law (to some extent) have begun to address drug- or alcohol-facilitated sexual assault. It took many decades for incapacitation to be considered a sexual assault perpetration tactic and that women’s behavior (e.g., voluntary alcohol consumption or consent while drunk) was not sufficient to absolve the responsibility of the person initiating sexual activity [173]. In fact, the FBI recently changed their definition of rape to include alcohol and/or drug use as a rape tactic and they also acknowledged that rape could occur without physical force [174].

Many scholars have already identified VSC as a sexual assault perpetration tactic and it is our hope that VSC should not only be considered on par with force or incapacitation, but that the presence of VSC should considered on its own, regardless of women’s response to such behavior. Going this direction may reflect the true reality and harm of sexual assault in that some research supports that verbal or emotional coercion produces similar personal consequences as physical force [49]. Focusing on a wide range of perpetration tactics would not only allow for a broader acknowledgement of the reality of sexual assault, but it also would remove the responsibility from the women involved and deemphasize that force or incapacitation is the only way the crime of rape can occur [47,88].

### 4.3. Toward a New Conceptualization of Unwanted Sex

We hope the evidence above supports our contention that consent resulting from VSC should be considered both as a result of and an indication of the lack of freely given consent, and therefore, placed as a tactic parallel to force [110,125,175]. With this outlook, we support the authors and legal jurisdictions that place verbal sexual coercion on par with sexual assault. Since researchers have also shown that unwanted sex can result from other factors, only focusing on VSC and its relationship to unwanted sex does not capture unwanted sex in all its complexity. As stated earlier, some scholars differentiate consent in situations where women consent due to prescribing to social norms and scripts, compliance or compromises to maintain relationships, and consent resulting from VSC. It appears that consent to unwanted sex resulting from societal pressure (e.g., it is my duty to have sex because I engaged in some consensual sex or invited him over) could be labeled either as compliance or as “problem sex” [50,103,140,142]. While the latter concept of problem sex points to concerning presence of and adherence to gender norms and sexual scripts, in situations where men do not use direct coercive tactics, these are not instances in which we can adequately state a rape occurred. Sexual compliance or compromises in the context of a relationship, if provided without historical or situational VSC, can be considered a benign compromise [46,67,95,101,114,169,170]. In situations where perpetration tactics are used, one can either maintain non-consent throughout the experience, or one may give in to sex, and neither of these possibilities should be considered freely given consent [118]. In this way, similar to Raghavan and colleagues [94], we maintain that non-consent could remain consistent throughout while consent not freely given, corresponds to their term of “forced compliance”, which referred to consent provided under duress (i.e., VSC).

**Figure 1:**
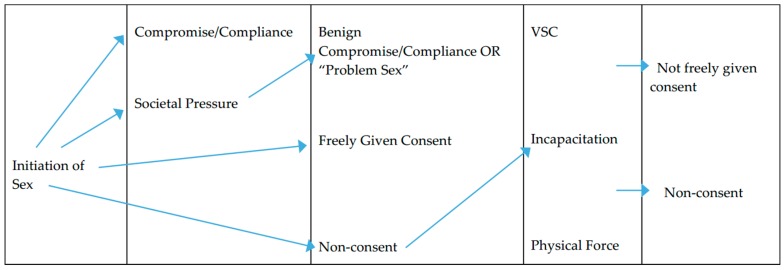
Relationship between coercion, compliance, and consent.

We have provided an illustrative chart (Figure 1) that begins with the initiation of sex and relates to five possible outcomes. To our knowledge, this is one of the first attempts to provide a conceptual model of consent that includes perpetration tactics, various types of unwanted sex, and the relationship between perpetration and consent identified in previous empirical research. In the chart below, the five outcomes are “freely given consent”, “non-consent”, “benign compromise/compliance”, “problem sex”, and “consent not freely given”. In situations where there is freely given consent, this refers to sex that is desired and where permission is granted to engage in sex and is free of VSC, incapacitation or physical force, and external societal pressure. There is an emphasis here that freely given consent includes desire because the other outcomes in this chart refer specifically to unwanted sex which could also include the lack of desire [114,150,168]. Non-consent refers to instances in which the person does not want to have sex and does not give their partner permission and this can refer to instances where all sexual activities end or it could be maintained throughout a sexual encounter where there are perpetration tactics present. Benign compromise/compliance can either be a result of maintaining relationships following the initiation of sex or from feeling external societal pressure. Problem sex refers to sex that occurs in the absence of perpetration tactics but due to societal pressure in which women may experience harm, but this does not reach the level of sexual assault. The last outcome refers to consent not freely given and these are the situations in which women consented to sex because they felt that they did not have a choice in the interaction since their partner did not respect their sexual refusal and instead engaged in perpetration tactics to obtain sex. 

It is important to note some relationships that are deliberately missing in this chart, particularly in our attempt, as much as possible, to be parsimonious with our conceptual argument. It is quite conceivable that there are interactions between each of these variables. First, it is possible that from the outset, the initiation of sex and freely given consent could be interlaced with societal pressure and expectations. It is also conceivable that there is a relationship between societal pressure and the use of perpetration tactics. For example, gender norms and sexual scripts hold that men are more likely to be, or should be, impersonal with sex, and impersonal sexual practices have been found to be related to the use of sexual aggression [176] and to psychopathic traits which also are associated with sexual assault perpetration [177,178]. Second, even though the arrows were not placed in this chart, we believe that VSC could be present by itself or with incapacitation and/or physical force, or that VSC could progress to one or both of those tactics [34,40,50,56,67,70,75,76,133,134,137,138,144,145,146,148,157,158]. Or as other previous research suggests, the use of physical force to obtain sex may be related to the future use of VSC in relationships [67]. Third, VSC or other perpetration tactics could be situational or there could be a pattern of coercion within a relationship leading to situational compliance to sex in the absence of immediate VSC [46]. Compliance in these situations could result from fear that if one does not comply, more aggressive tactics would be used [56,94]. This is not shown in the chart below. Fourth, some research has found that women that experienced sexual assault in the past or negative sexual encounters may be more likely to comply to unwanted sex from the outset [62,123,131,140], and this is a phenomena separate from consent from societal pressure and is not represented in the chart below. Last, with the empirical and conceptual scholarly work we reviewed in this paper, we do not believe that VSC could lead to outcomes other than maintained non-consent or consent not freely given. As we stated at the beginning of the paper, we sacrificed an in-depth analysis of the literature to cover a wide range of topics. As such, it is possible with a deeper analysis of these works that VSC, on its own, might lead to different outcomes presented or not presented here. Given these caveats, the following chart is not entirely complete. More research will further solidify these arguments and should be built upon and/or changed as more research is conducted and analyzed. Nonetheless, we believe this is a starting point. Overall, we contend that this conceptual chart could provide a way to critically think about unwanted sex and the relationship between consent and VSC and other perpetration tactics, which we believe has direct implications on the use of and potential efficacy of affirmative consent standards.

## 5. Discussion

The current article undertook multiple tasks. This paper outlined the ways in which sexual coercion, specifically VSC, as well as sexual consent, have been defined, operationalized, and studied in previous literature. This paper included the prevalence of VSC among college men and women from women’s reports of how often they experience VSC, consent to unwanted sex as a result of situational or historical VSC, and men’s reports of perpetrating VSC. Then, empirical evidence on women’s (non-)consent to unwanted sex as a result of VSC and men’s response to non-consent was presented. This was followed by a discussion on consent to unwanted sex as harmful consensual sex, harmless compromise, or not freely given consent largely based on the presence of VSC. Finally, we tied these arguments together and provided a chart to illustrate a potential way to conceptualize unwanted sex.

Not only was this paper important due to the relative lack of research and inconsistency on these topics and how they are related, but also due to the contemporary concerns of and policy changes taking place to address campus sexual assault. The following discussion will overview five consistent themes either underlying the arguments outlined or highlighted in this paper, followed by a brief discussion on the implications of adopting an affirmative consent standard, and a brief discussion of potential directions for future research, followed by a few concluding remarks.

Throughout this paper, some major themes emerged: (1) sexual assault is the result of gender imbalances in society, with men being the majority of perpetrators and women being primarily victims, (2) college campuses are areas where there is a high risk of sexual assault victimization and perpetration, (3) VSC is a tactic used by men to obtain sex from unwilling partners and can escalate to physical force, (4) negotiations of sexual (non-)consent are interpreted differently based on gender and within the broader society that endorses traditional gender norms and sexual scripts, and (5) consent resulting from VSC should not be considered freely given consent and should be considered a tactic parallel to force (e.g., sexual misconduct/criminal). Each of these themes will be briefly reviewed*.*

### 5.1. Sexual Assault Is the Result of Gender Imbalances in Society

Historically, men participated in the public (and political) sphere and represented the interests that state intervention could and would address. Therefore, law was created by men without female representation, in light of men’s economic and political interests, and based on cultural ideologies entrenched in a patriarchal society [54]. Historically, early conceptions of rape law ignored women’s sexual autonomy illustrated by the fact that the crime of rape was not a crime against the female victim, but rather a crime to her father, husband, or brother [54,109]. As such, it is not surprising then that rape is a crime well documented to be primarily against women, by men, making gender the most powerful predictor of rape [179] and although men can be and are victims of sexual violence, they are almost always the perpetrators [180].

### 5.2. College Campuses Are Places Where There Is a High Risk of Sexual Assault

While not all of the literature on consent and VSC covered in this paper was particular to college students, college students are part of the broader society in which gender norms, sexual scripts, and power dynamics govern heterosexual relations. College campuses are particular locations in which these norms, scripts, and dynamics are heavily endorsed, specifically within the alcohol-fueled hook-up culture [107]. What is more, college campuses are areas that comprise of some men that are willing to commit sexual assault through tactics involving verbal coercion, alcohol, or force, where some women are likely to defer to men and unlikely to reject sexual advances outright, and in situations where heavy alcohol use is involved and men are in control [21,51,52]. At the same time, men that report feeling entitled or have no moral disregard for committing sexual assault are not deterred from committing acts of sexual assault [154,181]. Certain groups on college campuses such as athletes or fraternities are supportive of sexual aggression and peer support for sexual assault is a predictor of sexual violence [144,145]. Of particular relevance, a fraternity at the University of Southern California that kept track of sexual conquests in 2010 made their message clear on how they felt about nonconsensual sex and rape: “non-consent and rape are two different things. There is a fine line, so make sure not to cross it” [77].

### 5.3. VSC Is a Tactic Used by Some Men to Obtain Sex from Unwilling Partners

Both men and women report that some men utilize coercive tactics, ranging from complimenting women and indicating how turned on they are, asking repeatedly, and trying to convince, or yelling/getting angry [34,38,46,49,56,67,75,88,113] to obtain sexual compliance. Studies have indicated or suggested that these types of behaviors may be a precursor to forcible rape [34,40,50,56,67,70,75,76,133,134,137,138,144,145,146,148,157,158]. Additionally, some men are aware that women are not consenting [48,50,127,134,144,145,146,150] and either proceed with sex regardless, use coercive tactics to obtain sex, or forego asking altogether if they think the answer would be no [50,120,133,168]. Taslitz [182] suggested that men utilize willful self-deception to justify their use of coercive tactics, and other studies have highlighted specific justifications men have used after committing sexual assault [148]. 

### 5.4. Negotiations of Sexual (Non-)Consent Are Affected by Gendered Norms and Sexual Scripts

Interestingly, the same gender norms and neoliberal ideas that women need to assert their sexual desires also appear to influence their view that verbal VSC is not a violation of sexual autonomy [132]. Both VSC and negotiations of consent should not be defined as gender neutral [53], as gendered norms, pervasive sexual scripts, and heterosexual power relations interact to create situations in which women do not think they can candidly say no to unwanted sex, perhaps because when they have said no, some men proceed with using coercive tactics to obtain sex despite knowing their partner is unwilling and then justify their behavior. As Jeffrey and Barata [75] pointed out in their study, a culture that condones rape, objectifies women, legitimizes sexual aggression, and places the responsibility of women to go against gender norms to articulate lack of consent in sexual situations, controls women in such a way that their sexual autonomy is undermined. Nonetheless, women are resilient in that some women may choose to consent to sex that they do not want in order not to be raped or experience additional harm, or they choose to interpret their experiences as something more benevolent than rape so as to avoid the victim label [34,46,48,50,56,91,102,104,131,132].

### 5.5. Consent Resulting from VSC Should Not Be Considered Freely Given Consent

Perhaps the largest goal we have attempted in this article is to examine closely the relationship between VSC and consent and with that showcase that consent resulting from VSC should be considered a lack of freely given consent. In fact, the Centers for Disease Control and Prevention state that sexual violence occurs when freely given consent is not present and they cite an “inability to refuse” as a result of numerous tactics, including intimidation or pressure [118]. It is our intention that conceptualizing consent as freely given or not could be used both within academic work, in the legal realm, and on college campuses. Considering the demarcation between sex and rape is the lack of consent, we contend in keeping with the literature that force is present due to the lack of consent, that verbal coercion is also present due to lack of consent, and both should be considered akin to rape. Vitiating consent as a result of coercion is in line with other ways in which the law renders consent ineffective in situations where it has been coerced, such as waiving Miranda Rights [110]. In addition, the Australian court system, when it comes to rape law, has stressed that holistic judgments of scenarios should be made to adequately determine if consent given by a victim was not freely and voluntarily given [183,184]. We have argued throughout this paper that consent as a result of societal pressure or desire to please a partner in an intimate relationship should not be considered rape if direct or past coercive tactics were not used to obtain consent.

Furthermore, it is worth noting the difference between consent and non-consent. If we recognize that nonconsensual sex is rape and that non-consent is represented by saying no, fighting, crying, pleading for him to stop, or as silence [113,114], then many women that ultimately consent to unwanted sex as a result of VSC have only done so after indicating non-consent. In this way, failing to acknowledge consent resulting from VSC as a lack of freely given consent also fails to acknowledge their initial non-consent. Ignoring initial non-consent in these situations treads closely to the idea that women should physically resist sex throughout the entire encounter to clearly demonstrate non-consent, which is an antiquated view that has been attacked by feminists and successfully removed as a requirement in sexual assault legal cases. It also highlights the idea that no *still* does not mean no, despite decades of rape awareness, prevention strategies and legal reform, begging the question as to whether sexual assault situations under a yes-means-yes standard would have any real impact on these situations and the responses to them. Recall the argument that VSC would not be present if consent was given initially. Therefore, if we focused on the final action of consent to unwanted sex, without taking into account that these women did not consent at the beginning of the sexual encounter and only consented after their partner did not take no for an answer, then we are failing to protect women’s sexual autonomy. Overall, acknowledging that consent resulting from VSC as not freely given and is both the result of and an indication of the lack of consent, has direct implications on an affirmative consent standard.

### 5.6. Affirmative Consent

Tuerkheimer [10] recently suggested that consent in general and on college campuses is the major issue at hand and is therefore also part of the solution of ameliorating the prevalence of rape in college campuses. Pressure from student activists and the federal government has resulted in a policy shift toward affirmative consent. Affirmative consent refers to a “yes-means-yes” standard and that initiators of sexual activity need to obtain an affirmative declaration and unequivocal yes to sexual activity in order for it to be considered consensual [19,20]. This type of standard rests on the notions that both men and women will be candid and explicit in communicating consent [19] and that the person initiating sexual activity would respect sexual refusals. Research suggests that some men may feel competent in sexual assertiveness (i.e., willing and able to engage in clear communication to obtain sexual consent), which was associated with interpreting sexual assault and sexual consent situations appropriately [147]. Yet, other men that endorse token resistance and rape myth acceptance, which are predictors of rape, had significant negative associations with interpretation of sexual consent in complex scenarios and rape myth acceptance was negatively associated with intentions to obtain consent [147]. Similarly, as indicated from the research outlined above, most women have a difficult time navigating the power dynamics inherent in heterosexual sex to say no to sex that they do not want, and in many cases are already providing consent to sex that they do not want sometimes as a result of VSC following sexual refusal. In addition, some men proceed with sex regardless of whether consent is provided, and may not even leave the space for women to provide consent [50,120]. These findings and the fact that we are moving from a “no-means-no” standard to a “yes-means-yes” standard, beg the question as to whether a policy change would have any effect on heterosexual encounters [22,23]. It may also create additional issues, such as holding women accountable for their own rape if it can be shown that she “consented”.

In fact, courts and administrators may not consider the power dynamics and coercive tactics used to obtain consent and instead focus solely on the fact that consent was provided, thereby downplaying or ignoring all the circumstances leading up to the “consent”. This would particularly occur in jurisdictions and at colleges that do not consider VSC as a problematic or criminal, and/or that do not define consent as “freely given”. As such, the debate on whether or not consent resulting from VSC should be considered null and the actions of the perpetrator criminal/sexual misconduct would only be amplified under an affirmative consent standard.

In this way, similar to the criticisms lodged at prevention programs for discounting the influence of the gendered power dynamics found in broader society and unique aspects of the campus culture [21], affirmative consent policies also can be critiqued in this light. These policies still maintain a focus on the person who is being asked for consent, which is typically the female as traditional gender roles have placed women as the sexual gatekeeper to indicate their (lack of) desire for sex [12,13,14,15]. Yet, this focus does not consider the broader societal structure in which these incidences take place, creating situations in which men feel entitled to sex, women feel they cannot say no to unwanted sex, and where some men do not take no for an answer. These policies also maintain focus on consent as the demarcation between sex and rape, and as outlined above, this renders consensual sex resulting from VSC a gray area.

Two ways to potentially address this would be to either decentralize consent when it comes to sex and rape [185] or focus on the actions of the perpetrator [46,109,171]. Echoing this, one study suggested that consent is not imperative to identify and conclude whether or not sexual coercion was present in the interaction [94]. These authors stated “it is more important to establish that sex was obtained under duress (i.e., that compliance was forced) regardless of how the victim resisted or showed (non-)consent at the time” [94] (p. 286; parentheses around “non-” was added). Similarly, as stated previously, some scholars, legal and otherwise, have argued that including coercive tactics as criminal or as sexual misconduct reflects the broader reality of sexual misconduct, and deemphasizes that force is the only way the crime of rape can be met [47]. The FBI recently amended the way they categorize rape and specifically indicated that they now include cases that do not involve physical force [174]. Similarly, the CDC has defined sexual violence as the lack of freely given consent and identified that one way this can occur is when the victim was unable to refuse because of intimidation or pressure [118]. If there is a concerted effort to protect women’s autonomy, then instances in which they consent to sex that is unwanted as a result of VSC should be considered as not freely given consent and perpetrators should be held accountable [46,186]. Other countries have begun to focus their response to violence against women in light of protecting women’s sexual autonomy and freedom from violence [187]. In order to do this, this would mean taking into account the circumstances in which she “consented” including examining the perpetrators behavior [94,169,183,184].

Some may argue that efforts to punish VSC through criminal prosecution or campus disciplinary procedures ignore existing legal requirements for proving intent. Specifically, it may be difficult for prosecutors or campus administrators to establish the alleged perpetrators’ mens rea in regards to VSC. Nonetheless, within rape law, there has been an evolution of changes that have paved the way for further adaptations to be made. For instance, rape law reformers have successfully been able to remove corroboration requirements, eliminate force and resistance requirements, enact rape shield laws, make marital rape illegal [188,189], include alcohol and/or drug use as a rape tactic [173,174], and in some cases make coercion a tactic parallel to force [110,125,175]. All of these changes indicate that moving in the direction of identifying VSC as a criminal act or sexual misconduct on college campuses may not be as impossible as it may seem.

What is more, a longstanding feminist critique directed at efforts to change individual behavior state that these efforts will continue to fail unless they focus on changing the broader culture, and in this case, also changing the campus culture [19,21,84]. Sexual violence is a complex problem that requires complex and comprehensive solutions [190]. The good news is that scholars and public health personnel are beginning to utilize ways, such as campus climate surveys [3] and environmental scans [6], to examine the campus culture as a whole to develop targeted interventions specific to individual campuses. This is one way in which unique factors that contribute to differences in rape-free and rape-prone campuses can be identified and addressed [98].

### 5.7. Future Research Directions to Consider

Based on the empirical and theoretical research examined in this article, there are several directions that future research can take, but only some are mentioned here. First, more research needs to assess the characteristics of men that utilize verbally coercive tactics and how and when these tactics proceed to additional coercive tactics and/or force. Second, research on VSC should be examined across nations and cultural contexts, as well as affording attention to ethnicity, race, and also non-heterosexual relations. Research should also continue to examine situations in which women use sexually coercive tactics, such as continual arguments and pressure following their partners’ sexual refusal. Third, one of the pressing issues discussed in this paper is that some women feel apprehensive in refusing sex from the outset, in many ways because of cultural norms or previous negative sexual encounters. Other women may consent out of fear of what may happen once non-consent has already been made clear and men are not taking no for an answer or because of previous patterns of coercion or force in relationships. What is more, some women report consenting to unwanted sex as a way to preserve their relationships or for other reasons that do not involve direct sexual perpetration tactics. More research should examine these different reasons and the historical, situational, and personal characteristics of these women consenting to unwanted sex. For example, some research has found a correlation between anxious attachments and compliance to sex, which suggests that women would consent to sex because they worry about the relationships or being up front [169]. Fourth, it is clear that more research needs to be conducted within both the social and legal realms to elucidate exactly how consent to unwanted sex should be conceptualized and addressed in law, scholarly work, as well as policies, prevention, and response to sexual violence on college campuses. For instance, perhaps this type of sexual assault should be considered as a lesser offense than forcible rape, but as an offense nonetheless, while scholars should continue researching and theorizing in this area, and prevention work should focus on ways to dismantle prevailing sexual scripts and gender norms that hold both men and women hostage. Last, moving forward, research needs to assess the utility of and possible unintended consequences of an affirmative consent standard in light of the empirical evidence provided in this paper and other relevant research.

## 6. Conclusions

Overall, campus sexual assault is a pervasive problem disrupting both the education and quality of life for all students. The complexity of consent and VSC is something scholars are only now beginning to truly unpack. As scholars move forward, one of the first steps is to come to a consensus on how to define and evaluate issues surrounding VSC and consent and the complex ways in which they are intertwined. Only then can scholars form an adequate understanding of these issues in order to provide suggestions on the best way to address campus sexual assault.

## Figures and Tables

**Table 1:** Research Gaps and Our Plan to Address Them.

Gap	Literature	How We Plan to Address the Gap
Definitions, Operationalizations, and Prevalence of (Verbal) Sexual Coercion	There is no consistent definition in the literature for sexual coercion or verbal sexual coercion in particular. This is despite that numerous studies have found that verbal sexual coercion is more common than other perpetration tactics [34,35,36,37,38,39,40,41,42,43,44,45,46,47,48,49,50]. Alcohol- and/or drug- involved rape, as distinct from other forms of rape, has been reviewed elsewhere [51,52], but reviews on sexual coercion and the different ways it has been operationalized and studied are lacking.	Discuss how (verbal) sexual coercion has been defined and operationalized. Then discuss the prevalence of verbal sexual coercion particularly in the college context.
Definitions and Negotiations of (Non-)Consent	There is no coherent or consistent definition of consent [32,53], despite the fact that consent is the demarcation between rape and sex and remains a central concern of rape law and policies as well as scholarly inquiry into the problem of rape [10,17,54].	Provide a brief overview of how consent has been defined and how (non-)consent is negotiated among college students.
Status of Unwanted Sex and the Relationship Between Verbal Sexual Coercion and Consent	Studies have not thoroughly discussed the relationship between consent and coercion. Over twenty years ago, Muehlenhard [55] highlighted the complexity surrounding both sexual coercion and consent and the relationship between them, and recently suggested that these issues remain today [32].	Discuss the relationship between verbal sexual coercion and consent. A conceptual chart combining all the concepts and relationships discussed in this paper is provided.
Potential Efficacy of an Affirmative Consent Standard	There has been a widespread adoption of an affirmative consent standard on college campuses, as this policy is considered to be an ideal response to CSA. However, affirmative consent policies have yet to be evaluated or critically considered as it pertains to reducing, preventing, and/or responding to CSA.	In light of the information presented in the previous sections, some implications of adopting affirmative consent standards will be discussed.

## References

[1] Schroeder L.P. (2013). Cracks in the ivory tower: How the Campus Sexual Violence Elimination Act can protect students from sexual assault. Loyola Univ. Chicago Law J..

[2] Carey K.B., Durney S.E., Shepardson R.L., Carey M.P. (2015). Incapacitated and forcible rape of college women: Prevalence across the first year. J. Adolesc. Health.

[3] Obama U.P. (2014). White House Task Force to Protect Students from Sexual Assault. Not Alone: Together against Sexual Assault.

[4] Krause K.H., Miedema S.S., Woofter R., Yount K.M. (2017). Feminist research with student activists: Enhancing campus sexual assault research. Fam. Relat..

[5] Fedina L., Holmes J.L., Backes B.L. (2018). Campus sexual assault: A systematic review of prevalence research from 2000 to 2015. Trauma Violence Abuse.

[6] McMahon S., Wood L., Cusano J., Macri L.M. (2018). Campus sexual assault: Future directions for research. Sex. Abuse.

[7] U.S. Department of Education, Office for Civil Rights (2001). Revised Sexual Harassment Guidance: Harassment of Student by School Employees, Other Students, or Third Parties. http://www.ed.gov/offices/OCR/archives/pdf/shguide.pdf.

[8] Karjane H.M., Fisher B.S., Cullen F.T. (2005). Sexual Assault on Campus: What Colleges and Universities are Doing about It.

[9] Richards T.N., Kafonek K. (2016). Reviewing state legislative agendas regarding sexual assault in higher education: Proliferation of best practices and points of caution. Fem. Criminol..

[10] Tuerkheimer D. (2015). Rape on and off Campus. Emory Law J..

[11] Antioch College (2016). Sexual Offense Prevention Policy (SOPP) Overview. https://www.antiochcollege.edu/sites/default/files/04.015_Sexual-Offense-Prevention-Policy.pdf.

[12] Fetterolf J.C., Sanchez D.T. (2015). The costs and benefits of perceived sexual agency for men and women. Arch. Sex. Behav..

[13] Dworkin S.L., O’Sullivan L. (2005). Actual versus desired initiation patterns among a sample of college men: Tapping disjunctures within traditional male sexual scripts. J. Sex Res..

[14] Wiederman M.W. (2005). The gendered nature of sexual scripts. Fam. J..

[15] Simms D.C., Byers E.S. (2013). Heterosexual daters’ sexual initiation behaviors: Use of the theory of planned behavior. Arch. Sex. Behav..

[16] Johnson A.M., Hoover S.M. (2015). The potential of sexual consent interventions on college campuses: A literature review on the barriers to establishing affirmative sexual consent. Pure Insights.

[17] Anderson M.J. (2016). Campus Sexual Assault Adjudication and Resistance to Reform. Yale Law J..

[18] Dripps D. (2008). After Rape Law: Will the Turn to Consent Normalize the Prosecution of Sexual Assault?. Akron Law Rev..

[19] Schulhofer S.J. (2015). Consent: What It Means and Why It‘s Time to Require It. Univ. Pac. Law Rev..

[20] Jozkowski K.N. (2015). “Yes means yes”? Sexual consent policy and college students. Chang. Mag. High. Learn..

[21] Armstrong A.E., Hamilton L., Sweeney B. (2006). Sexual assault on campus: A multilevel, integrative approach to party rape. Soc. Probl..

[22] Bay-Cheng L.Y. (2015). The agency line: A neoliberal metric for appraising young women’s sexuality. Sex Roles.

[23] Baker K.K., Oberman M. (2016). Women’s Sexual Agency and the Law of Rape in the 21st Century. Special Issue: Feminist Legal Theory.

[24] Fisher B.S., Cullen F.T., Turner M.G. (2000). The Sexual Victimization of College Women (NCJ 182369).

[25] Catalano S.M. (2004). Criminal Victimization, 2003.

[26] Black M.C., Basile K.C., Breiding M.J., Smith S.G., Walters M.L., Merrick M.T., Stevens M.R. (2011). The National Intimate Partner and Sexual Violence Survey (NISVS): 2010 Summary Report.

[27] Breiding M.J., Smith S.G., Basile K.C., Walters M.L., Chen J., Merrick M.T. (2014). Prevalence and characteristics of sexual assault, stalking, and intimate partner violence victimization: National intimate partner and sexual assault survey, United States, 2011. Morb. Mortal. Wkly. Rep. Surveill. Summ..

[28] Frazier P.A., Seales L.M., Schwartz M.D. (1997). Acquaintance rape is real rape. Researching Sexual Violence against Women.

[29] Wilson L.C., Miller K.E. (2016). Meta-analysis of the prevalence of unacknowledged rape. Trauma Violence Abuse.

[30] Cleere C., Lynn S.J. (2013). Acknowledged versus unacknowledged sexual assault among college women. J. Interpers. Violence.

[31] Dardis C.M., Kraft K.M., Gidycz C.A. (2017). “Miscommunication” and undergraduate women’s conceptualizations of sexual assault: A qualitative analysis. J. Interpers. Violence.

[32] Muehlenhard C.L., Humphreys T.P., Jozkowski K.N., Peterson Z.D. (2016). The complexities of sexual consent among college students: A conceptual and empirical review. J. Sex Res..

[33] Brown A.L., Testa M., Messman-Moore T.L. (2009). Psychological consequences of sexual victimization resulting from force, incapacitation, or verbal coercion. Violence Women.

[34] Edwards K.M., Probst D.R., Tansill E.C., Dixon K.J., Bennett S., Gidycz C.A. (2014). In their own words: A content-analytic study of college women’s resistance to sexual assault. J. Interpers. Violence.

[35] Holmgreen L., Oswald D.L. (2017). Men’s Sexual Coerciveness, Perceptions of Women’s Attachment, and Dating Preferences. Violence Vict..

[36] Strang E., Peterson Z.D., Hill Y.N., Heiman J.R. (2013). Discrepant responding across self-report measures of men’s coercive and aggressive sexual strategies. J. Sex Res..

[37] Abbey A., Wegner R., Pierce J., Jacques-Tiura A.J. (2012). Patterns of sexual aggression in a community sample of young men: Risk factors associated with persistence, desistance, and initiation over a 1-year interval. Psychol. Violence.

[38] French B.H., Suh H.N., Arterberry B. (2017). Exploratory factor analysis and psychometric properties of the sexual coercion inventory. J. Sex Res..

[39] Daspe M.È., Sabourin S., Godbout N., Lussier Y., Hébert M. (2016). Neuroticism and men’s sexual coercion as reported by both partners in a community sample of couples. J. Sex Res..

[40] Ramisetty-Mikler S., Caetano R., McGrath C. (2007). Sexual aggression among White, Black, and Hispanic couples in the US: Alcohol use, physical assault and psychological aggression as its correlates. Am. J. Drug Alcohol Abuse.

[41] Mouilso E.R., Calhoun K.S., Rosenbloom T.G. (2013). Impulsivity and sexual assault in college men. Violence Vict..

[42] Widman L., Olson M.A., Bolen R.M. (2013). Self-reported sexual assault in convicted sex offenders and community men. J. Interpers. Violence.

[43] Casey E.A., Masters N.T., Beadnell B., Hoppe M.J., Morrison D.M., Wells E.A. (2017). Predicting sexual assault perpetration among heterosexually active young men. Violence Women.

[44] Gilmore A.K., Schacht R.L., George W.H., Davis K.C., Norris J., Heiman J.R. (2014). Sexually transmitted infections risks among victims of sexual abuse and violence. J. Aggress. Maltreat. Trauma.

[45] Abbey A., BeShears R., Clinton-Sherrod A.M., McAuslan P. (2004). Similarities and differences in women’s sexual assault experiences based on tactics used by the perpetrator. Psychol. Women Q..

[46] Katz J., Tirone V. (2010). Going along with it: Sexually coercive partner behavior predicts dating women’s compliance with unwanted sex. Violence Women.

[47] Estrich S. (1987). Real Rape.

[48] Struckman-Johnson C., Struckman-Johnson D., Anderson P.B. (2003). Tactics of sexual coercion: When men and women won’t take no for an answer. J. Sex Res..

[49] Broach J.L., Petretic P.A. (2006). Beyond traditional definitions of assault: Expanding our focus to include sexually coercive experiences. J. Fam. Violence.

[50] Crown L., Roberts L.J. (2007). Against their will: Young women‘s nonagentic sexual experiences. J. Soc. Pers. Relatsh..

[51] Abbey A. (2002). Alcohol-related sexual assault: A common problem among college students. J. Stud. Alcohol Suppl..

[52] Lorenz K., Ullman S.E. (2016). Alcohol and sexual assault victimization: Research findings and future directions. Aggress. Violent Behav..

[53] Beres M.A. (2007). “Spontaneous” sexual consent: An analysis of sexual consent literature. Fem. Psychol..

[54] Bumiller K. (1988). The Civil Rights Society: The Social Construction of Victims.

[55] Muehlenhard C.L. (1996). The complexities of sexual consent. Siecus Rep..

[56] Livingston J.A., Buddie A.M., Testa M., VanZile-Tamsen C. (2004). The role of sexual precedence in verbal sexual coercion. Psychol. Women Q..

[57] Katz J., Wigderson S., Paludi M.A. (2012). “Put out or get out”: Understanding young women’s experiences of verbal sexual coercion by male dating partners. The Psychology of Love.

[58] Post L.A., Biroscak B.J., Barboza G., White J.W., Koss M.P., Kazdin A.E. (2011). Prevalence of sexual violence. Violence against Women and Children.

[59] DeGue S., DiLillo D. (2005). “You would if you loved me”: Toward an improved conceptual and etiological understanding of nonphysical male sexual coercion. Aggress. Violent Behav..

[60] Cook S.L., Gidycz C.A., Koss M.P., Murphy M. (2011). Emerging issues in the measurement of rape victimization. Violence Women.

[61] Krebs C. (2014). Measuring sexual victimization: On what fronts is the jury still out and do we need it to come in?. Trauma Violence Abuse.

[62] Davis K.C., Gilmore A.K., Stappenbeck C.A., Balsan M.J., George W.H., Norris J. (2014). How to score the Sexual Experiences Survey? A comparison of nine methods. Psychol. Violence.

[63] Krebs C.P., Lindquist C.H., Warner T.D., Fisher B.S., Martin S.L. (2007). The Campus Sexual Assault (CSA) Study: Final Report.

[64] Muehlenhard C.L., Goggins M.F., Jones J.M., Satterfield A.T. (1991). Sexual violence and coercion in close relationships. Sexuality in Close Relationships.

[65] Young B.R., Desmarais S.L., Baldwin J.A., Chandler R. (2016). Sexual coercion practices among undergraduate male recreational athletes, intercollegiate athletes, and non-athletes. Violence Women.

[66] Richardson E.W., Simons L.G., Futris T.G. (2017). Linking Family-of-Origin Experiences and Perpetration of Sexual Coercion: College Males’ Sense of Entitlement. J. Child Fam. Stud..

[67] Camilleri J.A., Quinsey V.L., Tapscott J.L. (2009). Assessing the propensity for sexual coaxing and coercion in relationships: Factor structure, reliability, and validity of the Tactics to Obtain Sex Scale. Arch. Sex. Behav..

[68] DeGue S., DiLillo D. (2004). Understanding Perpetrators of Nonphysical Sexual Coercion: Characteristics of Those Who Cross the Line.

[69] DeGue S., DiLillo D., Scalora M. (2010). Are all perpetrators alike? Comparing risk factors for sexual coercion and aggression. Sex. Abuse.

[70] Bushman B.J., Bonacci A.M., Van Dijk M., Baumeister R.F. (2003). Narcissism, sexual refusal, and aggression: Testing a narcissistic reactance model of sexual coercion. J. Personal. Soc. Psychol..

[71] Thompson M.P., Koss M.P., Kingree J.B., Goree J., Rice J. (2011). A prospective mediational model of sexual aggression among college men. J. Interpers. Violence.

[72] Hines D.A. (2007). Predictors of sexual coercion against women and men: A multilevel, multinational study of university students. Arch. Sex. Behav..

[73] Eaton A.A., Matamala A. (2014). The relationship between heteronormative beliefs and verbal sexual coercion in college students. Arch. Sex. Behav..

[74] McNulty J.K., Widman L. (2013). The implications of sexual narcissism for sexual and marital satisfaction. Arch. Sex. Behav..

[75] Jeffrey N.K., Barata P.C. (2016). “He Didn’t Necessarily Force Himself Upon Me, But...”: Women’s Lived Experiences of Sexual Coercion in Intimate Relationships With Men. Violence Women.

[76] Felson R.B. (2002). Violence and Gender Reexamined.

[77] Harned M.S. (2005). Understanding women’s labeling of unwanted sexual experiences with dating partners: A qualitative analysis. Violence Women.

[78] Brennan S., Statistics Canada (2011). Self-reported spousal violence, 2009. Family Violence in Canada: A Statistical Profile.

[79] World Health Organization/London School of Hygiene and Tropical Medicine (2010). Preventing Intimate Partner and Sexual Violence against Women: Taking Action and Generating Evidence.

[80] Lawyer S., Resnick H., Bakanic V., Burkett T., Kilpatrick D. (2010). Forcible, drug-facilitated, and incapacitated rape and sexual assault among undergraduate women. J. Am. Coll. Health.

[81] Schatzel-Murphy E.A., Harris D.A., Knight R.A., Milburn M.A. (2009). Sexual coercion in men and women: Similar behaviors, different predictors. Arch. Sex. Behav..

[82] Messman-Moore T.L., Coates A.A., Gaffey K.J., Johnson C.F. (2008). Sexuality, substance use, and susceptibility to victimization: Risk for rape and sexual coercion in a prospective study of college women. J. Interpers. Violence.

[83] Koss M.P., Abbey A., Campbell R., Cook S., Norris J., Testa M., White J. (2007). Revising the SES: A collaborative process to improve assessment of sexual aggression and victimization. Psychol. Women Q..

[84] Adams-Curtis L.E., Forbes G.B. (2004). College women’s experiences of sexual coercion: A review of cultural, perpetrator, victim, and situational variables. Trauma Violence Abuse.

[85] Faulkner G.E., Kolts R.L., Hicks G.F. (2008). Sex role ideology, relationship context, and response to sexual coercion in college females. Sex Roles.

[86] Kahn A.S., Jackson J., Kully C., Badger K., Halvorsen J. (2003). Calling it rape: Differences in experiences of women who do or do not label their sexual assault as rape. Psychol. Women Q..

[87] Thomas L.A., Gorzalka B.B. (2013). Effect of sexual coercion proclivity and cognitive priming on sexual aggression in the laboratory. J. Sex Res..

[88] Katz J., Moore J.A., Tkachuk S. (2007). Verbal sexual coercion and perceived victim responsibility: Mediating effects of perceived control. Sex Roles.

[89] Testa M., Dermen K.H. (1999). The differential correlates of sexual coercion and rape. J. Interpers. Violence.

[90] Koss M.P., Oros C.J. (1982). Sexual experiences survey: A research instrument investigating sexual aggression and victimization. J. Consult. Clin. Psychol..

[91] Gavey N. (1992). Technologies and effects of heterosexual coercion. Fem. Psychol..

[92] Finkelhor D., Yllo K. (1985). License to Rape.

[93] O’Sullivan L.F., Allgeier E.R. (1998). Feigning sexual desire: Consenting to unwanted sexual activity in heterosexual dating relationships. J. Sex Res..

[94] Raghavan C., Cohen S., Tamborra T. (2015). Development and preliminary validation of the multidimensional sexual coercion questionnaire (MSCQ). J. Sex. Aggress..

[95] Vannier S.A., O’Sullivan L.F. (2010). Sex without desire: Characteristics of occasions of sexual compliance in young adults’ committed relationships. J. Sex Res..

[96] Koss M.P., Gidycz C.A., Wisniewski N. (1987). The scope of rape: Incidence and prevalence of sexual aggression and victimization in a national sample of higher education students. J. Consult. Clin. Psychol..

[97] Haugen M.S. (2004). Does it matter what you call it? The relationship between labeling unwanted sexual experiences and distress. J. Consult. Clin. Psychol..

[98] Sanday P.R. (1996). Rape-prone versus rape-free campus cultures. Violence Women.

[99] Haugen A.D., Rieck S.M., Salter P.S., Phillips N.L. (2018). What makes it rape? A lay theories approach to defining rape among college students. Basic Appl. Soc. Psychol..

[100] Kalof L. (2000). Vulnerability to sexual coercion among college women: A longitudinal study. Gend. Issues.

[101] Walker S.J. (1997). When ‘no’ become ‘yes’: Why girls and women consent to sex. Appl. Prev. Psychol..

[102] Gavey N., Gergen M.N., Davis S.N. (1997). Feminist Poststructuralism and Discourse Analysis. Toward a New Psychology of Gender: A Reader.

[103] West R., Soble A. (2002). The Harms of Consensual Sex. The Philosophy of Sex: Contemporary Readings.

[104] Gavey N. (2005). Just Sex: The Cultural Scaffolding of Rape.

[105] Quinn-Nilas C., Kennett D.J. (2018). Reasons why undergraduate women comply with unwanted, non-coercive sexual advances: A serial indirect effect model integrating sexual script theory and sexual self-control perspectives. J. Soc. Psychol..

[106] MacKinnon C.A. (1987). Feminism Unmodified: Discourses on Life and Law.

[107] Bogle K.A. (2008). Hooking Up: Sex, Dating, and Relationships on Campus.

[108] Schulhofer S.J. (1998). Unwanted Sex: The Culture of Intimidation and the Failure of Law.

[109] Ryan K.M. (2011). The relationship between rape myths and sexual scripts: The social construction of rape. Sex Roles.

[110] Hong K.E. A New Mens Rea for Rape: More Convictions and Less Punishment. https://ssrn.com/abstract=3060709.

[111] Ford J.V. (2017). Sexual assault on college hookups: The role of alcohol and acquaintances. Sociol. Forum.

[112] Brownmiller S. (1975). Against Our Will: Men, Women, and Rape.

[113] French B.H., Neville H.A. (2017). What is nonconsensual sex? Young women identify sources of coerced sex. Violence Women.

[114] McGregor J. (2005). Is it Rape? On Acquaintance Rape and Taking Women’s Consent Seriously.

[115] Hickman S.E., Muehlenhard C.L. (1999). By the semi-mystical appearance of a condom: How young women and men communicate sexual consent in heterosexual situations. J. Sex Res..

[116] Clough A. (2018). Conditional Consent and Purposeful Deception. J. Crim. Law.

[117] Herring J. (2005). Mistaken Sex.

[118] Basile K.C., Smith S.G., Breiding M.J., Black M.C., Mahendra R.R. (2014). Sexual Violence Surveillance: Uniform Definitions and Recommended Data Elements (Version 2.0).

[119] Hall D.S. (1998). Consent for sexual behavior in a college student population. Electron. J. Hum. Sex..

[120] Jozkowski K.N., Peterson Z.D. (2013). College students and sexual consent: Unique insights. J. Sex Res..

[121] Conroy N.E., Krishnakumar A., Leone J.M. (2015). Reexamining issues of conceptualization and willing consent: The hidden role of coercion in experiences of sexual acquiescence. J. Interpers. Violence.

[122] Bay-Cheng L.Y., Eliseo-Arras R.K. (2008). The making of unwanted sex: Gendered and neoliberal norms in college women’s unwanted sexual experiences. J. Sex Res..

[123] Katz J., Schneider M.E. (2015). (Hetero) sexual compliance with unwanted casual sex: Associations with feelings about first sex and sexual self-perceptions. Sex Roles.

[124] Simon W., Gagnon J.H. (1986). Sexual scripts: Permanence and change. Arch. Sex. Behav..

[125] Fraser C. (2015). From Ladies First to Asking for It: Benevolent Sexism in the Maintenance of Rape Culture. Cal. Law Rev..

[126] Morgan E., Johnson I., Sigler R. (2006). Gender differences in perceptions for women’s participation in unwanted sexual intercourse. J. Crim. Justice.

[127] O’Byrne R., Rapley M., Hansen S. (2006). ‘You Couldn’t Say “No”, Could You?’: Young Men’s Understandings of Sexual Refusal. Fem. Psychol..

[128] Muehlenhard C.L., Hollabaugh L.C. (1988). Do women sometimes say no when they mean yes? The prevalence and correlates of women's token resistance to sex. J. Personal. Soc. Psychol..

[129] O’Byrne R., Hansen S., Rapley M. (2008). If a girl doesn't say ‘no’…”: Young men, rape and claims of ‘insufficient knowledge. J. Commun. Appl. Soc. Psychol..

[130] Shotland R.L., Hunter B.A. (1995). Women’s token resistant and compliant sexual behaviors are related to uncertain sexual intentions and rape. Personal. Soc. Psychol. Bull..

[131] Weiss K.G. (2009). “Boys will be boys” and other gendered accounts: An exploration of victims’ excuses and justifications for unwanted sexual contact and coercion. Violence Women.

[132] Burkett M., Hamilton K. (2012). Postfeminist sexual agency: Young women’s negotiations of sexual consent. Sexualities.

[133] Cook N.K., Messman-Moore T.L. (2018). I said no: The impact of voicing non-consent on women’s perceptions of and responses to rape. Violence Women.

[134] Power K. (2012). Luckily he backed off: A mixed methods analysis of undergraduate women’s consent, attitudes and behaviors. Columbia Soc. Work Rev..

[135] Kernsmith P.D., Kernsmith R.M. (2009). Gender differences in responses to sexual coercion. J. Hum. Behav. Soc. Environ..

[136] Abbey A., Wegner R., Woerner J., Pegram S.E., Pierce J. (2014). Review of survey and experimental research that examines the relationship between alcohol consumption and men’s sexual aggression perpetration. Trauma Violence Abuse.

[137] Katz J., Carino A., Hilton A. (2002). Perceived verbal conflict behaviors associated with physical aggression and sexual coercion in dating relationships: A gender-sensitive analysis. Violence Vict..

[138] Murphy C.M., Blumenthal D.R. (2000). The mediating influence of interpersonal problems on the intergenerational transmission of relationship aggression. Pers. Relatsh..

[139] MacKinnon C.A. (1989). Toward a Feminist Theory of the State.

[140] Bay-Cheng L.Y., Bruns A.E. (2016). Yes, but: Young women’s views of unwanted sex at the intersection of gender and class. Psychol. Women Q..

[141] Gross A.M., Winslett A., Roberts M., Gohm C.L. (2006). An examination of sexual violence against college women. Violence Women.

[142] Thomas E.J., Stelzl M., Lafrance M.N. (2017). Faking to finish: Women’s accounts of feigning sexual pleasure to end unwanted sex. Sexualities.

[143] Lisak D., Miller P.M. (2002). Repeat rape and multiple offending among undetected rapists. Violence Vict..

[144] Schwartz M.D., DeKeseredy W.S. Sexual Assault on the College Campus: The Role of Male Peer Support.

[145] DeKeseredy W.S., Schwartz M.D. (1993). Male peer support and woman abuse: An expansion of DeKeseredy‘s model. Sociol. Spectr..

[146] Beres M. (2010). Sexual miscommunication? Untangling assumptions about sexual communication between casual sex partners. Cult. Health Sex..

[147] Shafer A., Ortiz R.R., Thompson B., Huemmer J. (2018). The role of hypermasculinity, token resistance, rape myth, and assertive sexual consent communication among college men. J. Adolesc. Health.

[148] Hipp T.N., Bellis A.L., Goodnight B.L., Brennan C.L., Swartout K.M., Cook S.L. (2017). Justifying sexual assault: Anonymous perpetrators speak out online. Psychol. Violence.

[149] Motley M.T. (2008). Unwanted Escalation of Sexual Intimacy. Studies in Applied Interpersonal Communication.

[150] Lofgreen A.M., Mattson R.E., Wagner S.A., Ortiz E.G., Johnson M.D. (2017). Situational and dispositional determinants of college men’s perception of women’s sexual desire and consent to sex: A factorial vignette analysis. J. Interpers. Violence.

[151] Hust S.J., Rodgers K.B., Bayly B. (2017). Scripting sexual consent: Internalized traditional sexual scripts and sexual consent expectancies among college students. Fam. Relat..

[152] Suarez E., Gadalla T.M. (2010). Stop blaming the victim: A meta-analysis on rape myth. J. Interpers. Violence.

[153] Osman S.L. (2003). Predicting men’s rape perceptions based on the belief that “no” really means “yes“. J. Appl. Soc. Psychol..

[154] Bouffard L.A. (2010). Exploring the utility of entitlement in understanding sexual aggression. J. Crim. Justice.

[155] Bondurant B., Donat P.L. (1999). Perceptions of women's sexual interest and acquaintance rape: The role of sexual overperception and affective attitudes. Psychol. Women Q..

[156] Jozkowski K.N., Hunt M. Who wants a quitter?… So you just keep trying”: How college students’ perceptions of sexual consent privilege men. Proceedings of the Annual Meeting of the Society for the Scientific Study of Sexuality.

[157] Katz J., Moore J.A., May P. (2008). Physical and sexual covictimization from dating partners: A distinct type of interpersonal abuse?. Violence Women.

[158] White J.W., Smith P.H. (2009). Covariation in the use of physical and sexual intimate partner aggression among adolescent and college-age men: A longitudinal analysis. Violence Women.

[159] Cascardi M., O’Leary K.D., Lawrence E.E., Schlee K.A. (1995). Characteristics of women physically abused by their spouses and who seek treatment regarding marital conflict. J. Consult. Clin. Psychol..

[160] Marshall A.D., Holtzworth-Munroe A. (2002). Varying forms of husband sexual aggression: Predictors and subgroup differences. J. Fam. Psychol..

[161] Parker B., McFarlane J., Soeken K., Torres S., Campbell D. (1993). Physical and emotional abuse in pregnancy: A comparison of adult and teenage women. Nurs. Res..

[162] Kiefer A.K., Sanchez D.T. (2007). Men’s sex-dominance inhibition: Do men automatically refrain from sexually dominant behavior?. Personal. Soc. Psychol. Bull..

[163] Swartout K.M., Koss M.P., White J.W., Thompson M.P., Abbey A., Bellis A.L. (2015). Trajectory analysis of the campus serial rapist assumption. JAMA Pediatr..

[164] Cannon C., Lauve-Moon K., Buttell F. (2015). Re-theorizing intimate partner violence through post-structural feminism, queer theory, and the sociology of gender. Soc. Sci..

[165] Muehlenhard C.L., Powch I.G., Phelps J.L., Giusti L.M. (1992). Definitions of rape: Scientific and political implications. J. Soc. Issues.

[166] West R. (2010). Sex, law and consent. The Ethics of Consent: Theory and Practice.

[167] Rubenfeld J. (2013). The riddle of rape-by-deception and the myth of sexual autonomy. Yale Law J..

[168] Peterson Z.D., Muehlenhard C.L. (2007). Conceptualizing the “wantedness” of women's consensual and nonconsensual sexual experiences: Implications for how women label their experiences with rape. J. Sex Res..

[169] Impett E.A., Peplau L.A. (2003). Sexual compliance: Gender, motivational, and relationship perspectives. J. Sex Res..

[170] Gentzler A.L., Kerns K.A. (2004). Associations between insecure attachment and sexual experiences. Pers. Relatsh..

[171] Messman-Moore T.L., Long P.J. (2003). The role of childhood sexual abuse sequelae in the sexual revictimization of women: An empirical review and theoretical reformulation. Clin. Psychol. Rev..

[172] Bachman R. (1993). Predicting the reporting of rape victimizations: Have rape reforms made a difference?. Crim. Justice Behav..

[173] DeMatteo D., Galloway M., Arnold S., Patel U. (2015). Sexual assault on college campuses: A 50-state survey of criminal sexual assault statutes and their relevance to campus sexual assault. Psychol. Public Policy Law.

[174] Uniform Crime Report (2013). Crime in the United States. https://ucr.fbi.gov/crime-in-the-u.s/2013/crime-in-the-u.s.-2013/rape-addendum/rape_addendum_final.

[175] Tracy C.E., Fromson T.L., Long J.G., Whitman C. (2012). Rape and Sexual Assault in the Legal System.

[176] Abbey A., Jacques-Tiura A.J., LeBreton J.M. (2011). Risk factors for sexual aggression in young men: An expansion of the confluence model. Aggress. Behav..

[177] Harris G.T., Rice M.E., Hilton N.Z., Lalumiere M.L., Quinsey V.L. (2007). Coercive and precocious sexuality as a fundamental aspect of psychopathy. J. Personal. Disord..

[178] Knight R.A., Guay J.P., Patrick C.J. (2006). The role of psychopathy in sexual coercion against women. Handbook of Psychopathy.

[179] Koss M.P., Goodman L.A., Browne A., Fitzgerald L.F., Keita G.P., Russo N.F. (1994). No Safe Haven: Male violence against Women at Home, at Work, and in the Community.

[180] Rozee P.D., Koss M.P. (2001). Rape: A century of resistance. Psychol. Women Q..

[181] Bachman R., Paternoster R., Ward S. (1992). The rationality of sexual offending: Testing a deterrence/rational choice conception of sexual assault. Law Soc. Rev..

[182] Taslitz A.E. (2005). Willfully blinded: On date rape and self-deception. Harv. J. Law Gend..

[183] Crowe J., Sveinsson L. (2017). Intimidation, Consent and the Role of Holistic Judgments in an Australian Rape Law. Univ. Wert. Austl. Law Rev..

[184] (1995). The Queen v. Paul Stuart Shaw. https://archive.sclqld.org.au/qjudgment/1994/QCA94-551.pdf.

[185] Gardner J. (2017). The opposite of rape. Oxf. J. Leg. Stud..

[186] Stark E. (2007). Coercive Control: How Men Entrap Women in Personal Life.

[187] Tolmie J.R. (2018). Coercive control: To criminalize or not to criminalize?. Criminol. Crim. Justice.

[188] Spohn C.C. (1999). The Rape Reform Movement: The Traditional Common Law and Rape Law Reforms. Jurimetrics.

[189] Spohn C.C., Horney J. (1992). Rape Law Reform: A Grassroots Revolution and Its Impact.

[190] Yung C.R. (2015). Rape Law Fundamentals. Yale J. Law Fem..

